# The diagnosis and management of Cushing's syndrome in pregnancy

**DOI:** 10.1111/jne.13118

**Published:** 2022-05-01

**Authors:** Ross Hamblin, Amy Coulden, Athanasios Fountas, Niki Karavitaki

**Affiliations:** ^1^ Institute of Metabolism and Systems Research, College of Medical and Dental Sciences University of Birmingham Birmingham UK; ^2^ Centre for Endocrinology, Diabetes and Metabolism Birmingham Health Partners Birmingham UK; ^3^ Department of Endocrinology, Queen Elizabeth Hospital University Hospitals Birmingham NHS Foundation Trust Birmingham UK

**Keywords:** adrenal adenoma, Cushing's syndrome; pregnancy, hypercortisolism, pituitary tumour

## Abstract

Endogenous Cushing's syndrome (CS) is rarely encountered during pregnancy. Clinical and biochemical changes in healthy pregnancy overlap with those seen in pregnancy complicated by CS; the diagnosis is therefore challenging and can be delayed. During normal gestation, adrenocorticotrophic hormone, corticotrophin‐releasing hormone, cortisol, and urinary free cortisol levels rise. Dexamethasone administration fails to fully suppress cortisol in pregnant women without CS. Localisation may be hindered by non‐suppressed adrenocorticotrophic hormone levels in a large proportion of those with adrenal CS; smaller corticotroph adenomas may go undetected as a result of a lack of contrast administration or the presence of pituitary hyperplasia; and inferior petrosal sinus sampling is not recommended given the risk of radiation and thrombosis. Yet, diagnosis is essential; active disease is associated with multiple insults to both maternal and foetal health, and those cured may normalise the risk of maternal–foetal complications. The published literature consists mostly of case reports or small case series affected by publication bias, heterogeneous definitions of maternal or foetal outcomes or lack of detail on severity of hypercortisolism. Consequently, conclusive recommendations, or a standardised management approach for all, cannot be made. Management is highly individualised: the decision for surgery, medical control of hypercortisolism or adoption of a conservative approach is dependent on the timing of diagnosis (respective to stage of gestation), the ability to localise the tumour, severity of CS, pre‐existing maternal comorbidity, and, ultimately, patient choice. Close communication is a necessity with the patient placed at the centre of all decisions, with risks, benefits, and uncertainties around any investigation and management carefully discussed. Care should be delivered by an experienced, multidisciplinary team, with the resources and expertise available to manage such a rare and challenging condition during pregnancy.

## Graphical abstract

This comprehensive review highlights the diagnostic and therapeutic challenges encountered when faced with the pregnant patient with Cushing's syndrome. Following an overview of the changes in the HPA axis encountered in healthy pregnancy, we provide a guide to the diagnosis, management, and complications associated with this rare scenario.

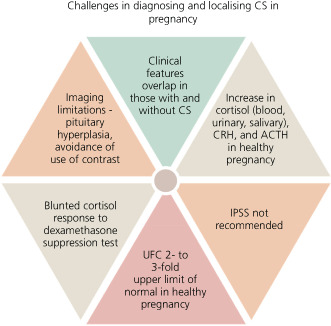

## 1 INTRODUCTION

Endogenous Cushing's syndrome (CS) is rare, with an estimated annual incidence of 1.2–3.2 cases per million in a population.^1^ Given that the archetypal patient with CS is a female of childbearing age, encountering CS in pregnancy may not seem to be such an unusual occurrence. However, menstrual irregularities are present in more than half of women with CS, and hypercortisolaemia impairs gonadal function at the level of the hypothalamus, pituitary gland, and ovaries.^2^ Thus, CS in pregnancy is extremely rare. Fewer than 250 cases have been reported, of which, less than 100 have received active treatment.^3^, ^4^, ^5^, ^6^ The diagnosis is challenged by a commonality of clinical features seen in both those with or without CS in healthy pregnancy, compounded by a lack of validated biochemical tests that can accurately discriminate between physiological activation of the hypothalamic‐pituitary‐adrenal axis (HPA) and pathological hypercortisolaemia.^7^, ^8^

Although systematic reviews on both CS^5^ and Cushing's disease (CD)^4^ in pregnancy have been published, given the condition's rarity, the evidence used to formulate such reviews is limited, consisting of case reports or small case series only.^9^, ^10^ Similarly, recent clinical practice guidelines on pituitary adenomas in pregnancy by the European Society of Endocrinology provide guarded recommendations for those with CS, mainly reliant on expert opinion and low‐quality evidence only.^3^ Thus, there is uncertainty regarding the optimum therapeutic strategy. Despite such limitations, the diagnosis and management of CS pregnancy is of major importance given the potential impact to both mother and foetus if left undiagnosed or managed inadequately.

Thus, in this review, which forms part of a special issue titled ‘Update of Cushing's Syndrome: 100 years after Minnie G’, we highlight the diagnostic and therapeutic challenges encountered when faced with the pregnant patient with CS. Following an overview of the changes in the HPA axis encountered in healthy pregnancy, we provide a guide regarding the diagnosis and management of this rare scenario, as well as the associated complications.

## 2 PHYSIOLOGICAL CHANGES TO THE HPA AXIS DURING PREGNANCY AND LABOUR

During pregnancy, there are substantial changes to the HPA axis, which becomes hyperactive, leading to a state of physiological hypercortisolism.^9^, ^11^ This is characterised by a rise in corticotrophin‐releasing hormone (CRH), adrenocorticotrophic hormone (ACTH), and free and total cortisol levels.^9^, ^11^, ^12^, ^13^

The placenta starts to release CRH (placental CRH) into the maternal bloodstream from the first trimester, but more significantly during the second and third trimesters,^12^, ^14^, ^15^ leading to a substantial rise in circulating plasma CRH of up to 1000‐fold.^16^ Placental CRH is molecularly identical to maternal CRH,^14^ and stimulates the maternal pituitary and subsequently the adrenal glands to secrete ACTH and cortisol, respectively.^11^, ^12^, ^13^ In late pregnancy, the maternal CRH production is downregulated in response to the increased cortisol levels, leading to desensitisation of corticotroph cells; this has been proposed as a potential mechanism explaining the attenuated response to CRH.^9^, ^12^ The rise in ACTH and cortisol during pregnancy is modest compared to the significant increase in CRH.^17^ This is partly as a result of changes in the CRH‐binding protein (CRH‐BP), which reduces the bioavailability of CRH. Levels of CRH‐BP are similar to those of non‐pregnancy during the first two trimesters, but, in the final trimester, they decrease considerably, and bioavailable plasmatic CRH is consequently elevated.^18^ The increase in CRH plays a role in the labour process and in foetal lung maturation.^11^, ^13^, ^14^ ACTH levels steadily escalate throughout pregnancy in a sawtooth pattern until the final weeks. During labour, levels markedly increase, peaking at delivery.^9^

With progressing gestation, there is a gradual rise in total circulating cortisol; the increase is significant by the end of first trimester (1.6‐fold),^13^ with steady rise to 2.4‐fold by the second, and up to 3‐fold non‐pregnant levels by the third trimester.^11^, ^12^, ^13^, ^19^, ^20^ This is primarily a result of the increased hepatic production of corticosteroid‐binding globulin (CBG),^12^ a consequence of elevated oestrogen levels encountered in pregnancy.^13^ CBG levels gradually rise during gestation,^11^, ^13^, ^20^ before falling in the final weeks of pregnancy, leading to increased free cortisol levels.^11^, ^13^ Free cortisol also increases during pregnancy as a result of changes in the activity of the HPA axis.^9^, ^12^ This has been demonstrated by several longitudinal studies^11^, ^13^, ^21^ showing a 3‐fold increase in both urinary free cortisol (UFC) in the third trimester^13^, ^21^ and 1.8‐fold rise in serum free cortisol between 16 and 36 weeks of gestation.^11^ Jung et al.^13^ found that UFC rose proportionately higher than serum free cortisol, likely as a result of an increased metabolic clearance of free cortisol during pregnancy. Night‐time salivary cortisol also rises progressively during pregnancy, reaching a peak of 2.1‐fold that of non‐pregnancy levels in the third trimester.^22^ However, diurnal variation in cortisol is still maintained, but the level of fluctuation from the mean becomes blunted.^9^, ^12^, ^21^

Despite a significant rise in maternal cortisol levels, the foetus is protected. Placental 11β‐hydroxysteroid dehydrogenase type 2 enzyme (HSD11B2) in the syncytial trophoblastic cells converts active cortisol to inactive cortisone.^9^, ^11^, ^12^, ^13^ This ensures that foetal cortisol levels remain lower than maternal ones; indeed, 80–90% of the circulating cortisol is metabolised by the placenta, preventing exposure to the foetus.^9^, ^12^ Such protective measures, however, are not full proof. Indeed, 25% of total foetal cortisol is maternal in origin at full‐term,^11^ with high maternal cortisol levels and foetal dependence on HSD11B2 activity influencing foetal exposure. Thus, even modest shifts in maternal cortisol, such as in periods of maternal stress from anxiety, inflammation or infection, can lead to excessive levels of cortisol reaching the foetus.^12^ Furthermore, the inactivation of cortisol by HSD11B2 is reversed in late pregnancy.^9^ Higher levels of cortisol during late pregnancy may help with late foetal development, such as lung maturation.^23^

The continual rise in placental CRH during gestation peaks 48 hours  prior to parturition, leading to a spike in ACTH release.^14^ It is postulated that this prepares the mother for the physical and metabolic stressor of labour and aids the final stages of foetal organ development.^11^, ^13^ The CRH peak at this point is suggestive of a possible role in stimulating labour.^24^ At the onset of labour and delivery, ACTH and cortisol levels also rise significantly.^9^, ^14^, ^19^ As expected with such an acute stressor, ACTH and cortisol concentrations are maximal, with ACTH 10‐fold that of non‐pregnant subjects during labour.^9^

After birth, the rapid withdrawal of placental CRH^12^ leads to reduction in CRH and ACTH levels as early as 2  hours post‐delivery.^14^, ^25^ This allows a gradual return to the normal physiological state of maternal HPA axis with a reduction in maternal cortisol, although this can be a slow process.^12^ Full resolution of normal CRH secretion requires at least 3 months post‐partum, whereas normal suppression of cortisol following dexamethasone may take up to 5 weeks after birth.^26^ There is no consensus on the rate at which CBG normalises to non‐pregnancy levels after birth, with a range of normalisation between 3 weeks and 3 months post‐delivery.^13^ Free cortisol levels are likely to normalise within a week of birth; however, the reported speed at which this occurs varies.^12^, ^13^, ^25^, ^27^ By 3 months after birth, both free cortisol and UFC have normalised.^13^, ^27^ However, total cortisol levels remain elevated at 2–3 months postpartum, likely as a result of continued elevation in CBG.^13^

A summary of the changes in the HPA axis during pregnancy are summarised in Table 1.

**TABLE 1:** Changes in the blood levels of total cortisol, corticosteroid‐binding globulin (CBG), free cortisol, adrenocorticotrophic hormone (ACTH), corticotrophin‐releasing hormone (CRH), and response to a 1‐mg dexamethasone suppression test at each trimester of pregnancy and 3 months post‐partum.^9^, ^28^

	Trimester			Labour and delivery	Three months post delivery
	First	Second	Third		
Total cortisol	↑	↑↑	↑↑↑	↑↑↑	↔
CBG	↑	↑↑	↑↑↑	↑↑	↔
Free cortisol	↑	↑↑	↑↑↑	↑↑↑↑	↔
ACTH	↑	↑↑	↑↑↑	↑↑↑↑	↔
CRH	↑	↑↑	↑↑↑	↑↑↑↑	↔
Response to 1 mg dexamethasone suppression test	↓	↓↓	↓↓	‐	↔

*Note*: ↑, ↓, and ↔ represent an increased level, decreased level, and the same level compared to pre‐pregnancy.

## 3 AETIOLOGY

**FIGURE 1:**
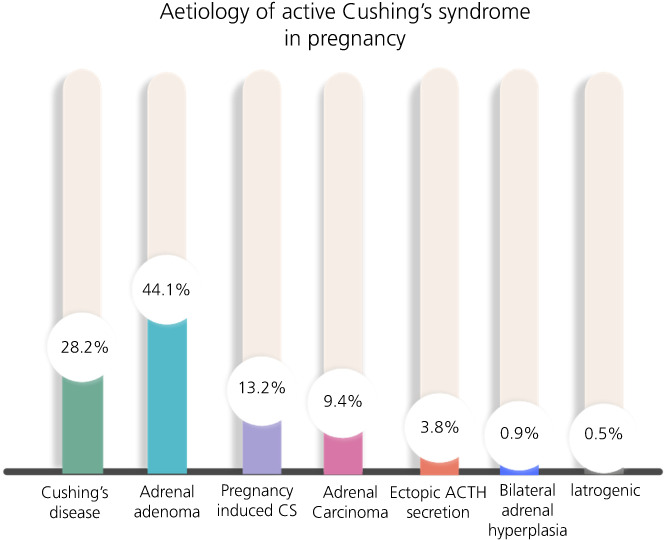
Aetiology of 213 gestations with active Cushing's syndrome (CS) (data reported by Caimari et al.^5^). Includes both those with active disease during pregnancy and those newly diagnosed with CS within 12 months of pregnancy. Abbreviations: ACTH, adrenocorticotrophic hormone

Any cause of CS encountered outside of pregnancy can arise during pregnancy, but the prevalence of such causes differs (Figure 1). Notably, in contrast to non‐pregnant subjects where CD is responsible for around 75% of CS,^29^ pathological hypercortisolaemia derived from an adrenal source (adrenal adenoma, bilateral macronodular adrenal hyperplasia, adrenocortical carcinoma or primary pigmented adrenal disease) is more commonly diagnosed in pregnancy (54% adrenal CS vs. 28% CD).^5^ Androgen hypersecretion is more likely in those with pituitary corticotroph adenomas and is a further barrier to conception for those with CD, possibly accounting for the changes in disease prevalence observed during pregnancy.^30^

Pregnancy‐induced CS,characterised by the transient manifestation of clinical and biochemical features of CS that typically remit following delivery, has been reported in a small number of cases.^31^, ^32^, ^33^, ^34^, ^35^, ^36^, ^37^, ^38^ This rare manifestation has been attributed to aberrant luteinising hormone receptors expressed in adrenal cortex, where hypercortisolaemia is induced by increased levels of HCG during pregnancy, in an ACTH‐independent manner.^34^, ^39^, ^40^, ^41^ CS in pregnancy may also be exacerbated by placental derived ACTH stimulation of melanocortin‐2 receptors, present in adrenocortical adenomas,^42^ or by rises in oestradiol during pregnancy, as reported in one patient with primary adrenocortical nodular hyperplasia.^34^, ^43^ Adrenal imaging may be normal, but the presence of adrenal adenoma, carcinoma, and bilateral micro‐ and macronodular hyperplasia, have all been reported in this condition.^31^

CS secondary to adrenocortical carcinoma (ACC), ectopic ACTH production or bilateral adrenal hyperplasia is rare.^44^, ^45^, ^46^, ^47^, ^48^, ^49^

## 4 CLINICAL FEATURES

There is significant overlap between symptoms and signs experienced in a healthy pregnancy and those in a pregnancy with CS. Weight gain, mood disturbance, acne, non‐violaceous striae, and facial plethora are commonly seen in healthy subjects and thus lack discriminative value. The appearance of violaceous striae (particularly in sites other than the abdomen), proximal myopathy, skin thinning, easy bruising, and pathological fractures are not typical of pregnancy however, and can be used as discriminatory manifestations.^3^ Co‐morbidities, including gestational diabetes and gestational hypertension, which can be helpful for diagnosis outside pregnancy, are less useful during pregnancy, given that they can complicate pregnancy in the absence of CS.^50^ However, previous medical and obstetric history is relevant. Women with CS are more likely to have a prior history of hypertension, type 2 diabetes, spontaneous abortion, and gestational diabetes. Furthermore, a history of pre‐eclampsia or foetal loss is 6‐ and 10‐fold more likely, respectively, in pregnancies complicated by CS compared to healthy pregnancies.^5^

## 5 BIOCHEMICAL CONFIRMATION OF CS

### 5.1 UFC

UFC measurement can be used for establishing the diagnosis of CS in pregnancy. Although UFC excretion does not change from the pre‐pregnancy status in the first trimester, values rise by 2‐ to 3‐fold in the second and third trimester in healthy pregnancy, and thus levels within this range are non‐discriminatory. Values greater than 3‐fold the upper normal reference range should be considered potentially indicative of CS.^8^

### 5.2 Overnight or low‐dose dexamethasone suppression test

Use of either the overnight dexamethasone suppression test or low‐dose dexamethasone suppression test is not recommended in pregnancy. In healthy pregnant women, there is attenuation of suppression of cortisol secondary to both increased levels of cortisol and of CBG. Indeed, following the administration of dexamethasone, blood cortisol has been shown to decrease by just 40.2% compared to a reduction of 87.4% in non‐gravid, healthy controls.^28^ Furthermore, the increase in the total cortisol as a result of a rise in CBG affects the reliability of the dexamethasone suppression tests. Subsequent high false positive rates thus limit their value for diagnosing pathological hypercortisolaemia during pregnancy.^8^

### 5.3 Late night salivary cortisol

Late night salivary cortisol (LNSC) levels are 2‐fold higher in normal pregnancy.^22^ However, the circadian rhythm is maintained, and thus healthy pregnant women demonstrate diurnal cortisol changes. By contrast, those with CS in pregnancy lose this physiological diurnal variation, a distinguishing discrepancy that can be identified using late night salivary cortisol measurement. Using the Salimetrics® Cortisol Enzyme Immunoassay kit (Salimetrics, LLC), Lopes et al.^22^ established trimester specific LNSC cut‐off values to discriminate between healthy subjects in pregnancy and those with CD in pregnancy. LNSC cut off values of 7.0, 7.2, and 7.9 nmol L^−1^ for the first, second, and third trimester respectively, had a sensitivity of 80%–92% and specificity of 93%–100% in the diagnosis of CD. Establishing cut‐offs for other assays is required.

## 6 LOCALISING THE SOURCE OF CS

### 6.1 Plasma ACTH

In pregnancy, non‐supressed ACTH cannot reliably exclude an adrenal source of CS. Indeed, in one series, 50% of patients with ACTH‐independent CS did not have a supressed ACTH.^6^ In pregnant patients with CD, ACTH levels are usually in the upper half of the normal range or above the upper reference range.^7^

### 6.2 CRH, desmopressin, and high‐dose dexamethasone suppression test

CRH administration fails to induce a significant rise in ACTH or cortisol in gravid, healthy subjects.^9^, ^51^ Evidence is limited, but CRH administration in five patients with CS during pregnancy induced an incremental cortisol rise of 44%–130%, consistent with CD later confirmed on histology. Thus, some advocate its use if ACTH‐independent CS is unlikely on initial testing.^6^ Human CRH has not been found to be teratogenic in animal studies, and ovine CRH can be given in pregnancy provided that it is clinically indicated.^9^

There are two case reports of desmopressin stimulation in the diagnostic work‐up of CD during pregnancy.^52^, ^53^ In the first one, ACTH rose by 70%, 15 min after 10 μg of desmopressin given at 14 weeks gestation.^53^ In the second, both desmopressin and CRH were given on separate occasions in a pregnant patient with suspected CD; desmopressin resulted in a higher incremental ACTH increase than that observed after CRH administration.^53^ Both cases later had transsphenoidal surgery (TSS) with corticotroph adenomas confirmed on histology.

The use of the high‐dose dexamethasone suppression test has been suggested by some, with a fall in cortisol of greater than 80% reported to distinguish between adrenal and pituitary source of CS in pregnancy.^6^, ^54^

As a result of the limited evidence available and the lack of data from normal pregnancies, the use of these three tests is generally not recommended in the setting of pregnancy.^3^, ^55^

### 6.3 Pituitary magnetic resonance imaging (MRI)

Pituitary microadenomas are responsible for more than 90% of CD.^8^ Tumour detection can therefore prove difficult; 40% of pituitary microadenomas are not detected by pituitary MRI and, when detected, they may be incidental, clinically irrelevant findings.^8^, ^56^ Although such challenges exist for all patients with CD (as covered by Balomenaki et al^57^ ‘Diagnostic workup for Cushing's syndrome’ in this special issue), diagnostic difficulties are accentuated in pregnancy. First, gadolinium use alongside MRI should be limited^58^ and, second, the pituitary gland undergoes hyperplasia and thus smaller tumours are even more difficult to detect.^59^ For patients with biochemical evidence of CD, but with absence of discernible adenoma on non‐contrast MRI pituitary imaging, careful discussion with the patient regarding the risks and benefits of further investigation should be advised. Gadolinium has been shown to cross the placenta, but, reassuringly, no mutagenic or teratogenic effects have been observed in human studies following administration during pregnancy.^58^ Current guidelines recommend use of contrast when it will lead to a change in maternal or foetal outcome, and investigation or treatment cannot be delayed until after the pregnancy.^60^

### 6.4 Bilateral inferior petrosal sinus sampling

Bilateral inferior petrosal sinus sampling (IPSS) is not advisable during pregnancy, given the associated risk of venous thromboembolism and radiation.^3^ Of the limited reported number of CD treated with TSS during pregnancy, some received IPSS prior to surgery, none of whom developed procedural complications.^6^, ^61^ Some have advocated the use of a direct jugular approach for venous catheterisation and lead barrier protection to shield the foetus from radiation^6^; however, given the limited evidence and associated complications, IPSS is not recommended in pregnancy.^3^

### 6.5 Adrenal imaging

In the case of low or normal ACTH levels, coupled with confirmed hypercortisolism, adrenal MRI without contrast should be performed to look for an adrenal lesion. Adrenal ultrasound may also be helpful in some cases.^3^ Given superior alternatives and potential risks associated with computed tomography (CT), a CT scan (with or without contrast) should not be performed in the diagnostic work‐up of CS in pregnancy.

## 7 MANAGEMENT

**FIGURE 2:**
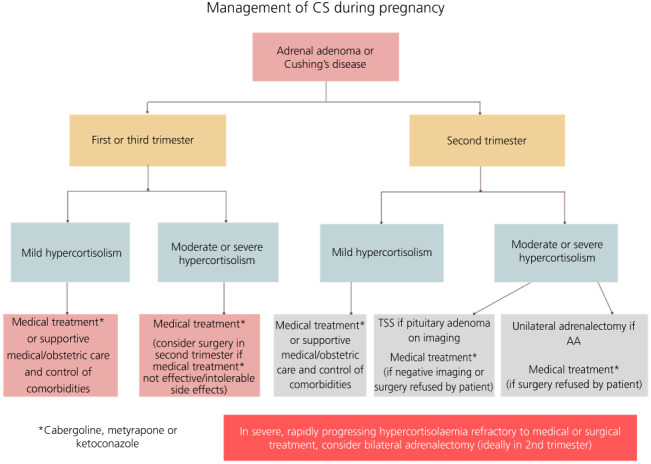
Treatment algorithm for management of Cushing's syndrome (CS) in pregnancy. Given limited evidence, this should be used as a guide only. Abbreviations: AA, adrenal adenoma; TSS, transsphenoidal surgery

Given a scarcity of evidence, no definitive conclusions on the superiority of available management approaches can be made for those who require treatment for CS during pregnancy. Treatment should be individualised, with a need to consider the patient's wishes, stage of pregnancy, severity of hypercortisolism, identification of pituitary adenoma on imaging (in cases of CD), existing maternal co‐morbidities, and risk of maternal‐foetal harm.^3^, ^7^, ^62^ Effective multidisciplinary team working is a necessity,^63^ with the patient placed at the centre of all decisions and managed in tertiary referral centres or centres of excellence, amongst clinicians with experience in managing endocrinopathy in pregnancy. Throughout the pregnancy, risks, benefits, and uncertainties around any investigation or treatment offered should be carefully discussed with the patient. A proposed management algorithm for CS in pregnancy is highlighted in Figure 2.

### 7.1 Conservative management

Given the risks to mother and foetus are highest for those untreated,^3^ active treatment, with either surgical or medical therapy, should be considered and discussed with all. However, for cases with mild disease, such as those with modest levels of hypercortisolaemia and without evidence of complications, conservative management may be a good option. Close monitoring, with management of hypercortisolaemic complications, including glucose, electrolytes, and blood pressure control, is essential. Thromboprophylaxis with low molecular weight heparin should be strongly considered given the thrombotic propensity of both pregnancy and CD.^3^, ^64^

### 7.2 Pituitary surgery

To date, there are just 14 case reports of TSS for the management of CD in pregnancy (published in English) (Table 2). Surgery should ideally be performed in the second trimester, thereby avoiding the potential risks of abortion and pre‐term labour that have been associated with an operation in the first and third trimester, respectively.^3^, ^7^, ^71^

**TABLE 2:** Summary of the published cases of transsphenoidal surgery performed during pregnancy for the management of Cushing's disease

Reference (Year)	Time of TSS (trimester, week of gestation)	Surgical complications	Maternal and foetal complications	Delivery	Reported outcome of Cushing's disease
Casson et al.^65^ (1987)	Second, 22 weeks	NR	Pre‐eclampsia at 28 weeks Foetal intubation and pneumothoraces	Emergency caesarean at 30 weeks	NK
Coyne et al.^66^ (1992)	Second, 14 weeks	Permanent DI	Nil	NK	Remission
Pinette et al.^61^ (1994)	Second, 16 weeks	NR	Intrauterine death	33 weeks, tight nuchal cord	Persistence of disease
Ross et al.^67^ (1995)	Second, 18 weeks	CSF leak, transient DI	Labile HTN (32 weeks) IUGR, foetal distress (37 weeks)	Induction of labour, emergency caesarean (37 weeks)	Remission
Mellor et al.^68^ (1998)	Second^a^	NR	Severe eclampsia (33 weeks) Low birth weight (2.3 kg)	Emergency caesarean under general anaesthesia (33 weeks)	Remission
Verdugo et al.^69^ (2004)	Second, 23 weeks	NR	Nil	39 weeks, vaginal delivery	Remission
Lindsay et al.^6^ (2005)					
*Case 1*	Second, 18 weeks	NR	Severe pre‐eclampsia IUGR, low birth weight (1.7 kg)	Induced vaginal labour, 34 weeks	Remission
*Case 2*	Second, 14 weeks	Transient SIADH	Intrauterine death	Stillborn delivered at 33 weeks, tight nuchal cord	Persistence of disease BAH and RT required 3 months post pregnancy
*Case 3*	First, 10 weeks +5 days	NR	NR	Vaginal delivery at term	Remission
*Case 4*	Second, 17 weeks	Transient SIADH	Persistent HTN, pre‐eclampsia Reversal of cord blood flow, foetal death 5 days after delivery	caesarean (at 24 weeks)	Remission
Boronat et al.^70^ (2010)	Second, 16 weeks	NR	Persistent HTN, gestational diabetes (24 weeks) Low birth weight (2.4 kg)	Induction of labour at 34 weeks	Persistence of disease, also had ketoconazole during first trimester and metyrapone for remainder of pregnancy
Abbassy et al.^*71*^ (2015)	Second, 18 weeks	Permanent DI	NR	Vaginal, 39 weeks	Remission
Jolly et al.^10^ (2019)	Second, 23 weeks	nil	HTN (33 weeks) Congenital diaphragmatic hernia, foetal death	Emergency caesarean section at 38 weeks	Remission
Sridharan et al.^4^ (2021)	Second, 20 weeks	nil	Vomiting and hypoglycaemia post‐operatively (before administration of hydrocortisone)	Vaginal delivery at 40 weeks	Remission

^a^ Week of second trimester not specified.

Abbreviations: BAH, bilateral adrenal hyperplasia; CSF, cerebrospinal fluid; DI, diabetes insipidus; HTN, hypertension; IUGR, intrauterine growth restriction; NK, not known; NR, not reported; RT, radiotherapy; SIADH, syndrome of inappropriate antidiuretic hormone secretion; TSS, transsphenoidal surgery.

Based on limited data assessed in a systematic review, TSS appears to be relatively safe and successful, with a remission rate of 76.9%.^4^ Surgical complications include cerebrospinal fluid leak in one patient (7.7%), transient syndrome of inappropriate diuretic hormone secretion in two patients (15.4%), transient diabetes insipidus (DI) in one patient (7.7%), and permanent DI in two patients (15.4%).^4^

From a practical perspective, surgery is ideally performed with the patient lying supine, tilted in the left lateral position to prevent reduced venous return, which may arise if the uterus compresses the inferior vena cava.^62^ Prophylactic anticoagulation before and after surgery should be strongly considered given increased risks of venous thromboembolism following surgery, exacerbated by the pregnant state and hypercortisolaemia.^64^, ^72^, ^73^

### 7.3 Medical therapy

Metyrapone,^31^, ^35^, ^70^, ^74^, ^75^, ^76^, ^77^, ^78^ ketoconazole,^70^, ^74^, ^79^, ^80^, ^81^ cabergoline,^80^, ^82^, ^83^, ^84^ mitotane,^85^, ^86^ cyproheptadine,^87^, ^88^, ^89^ and aminogluthemide^5^, ^6^ have all been used in the management of patients with CS during pregnancy. Each is discussed in turn, along with their benefits and limitations, in Table 3. Further information regarding the medical management of CD outside of pregnancy is covered by Castinetti^90^ in: ‘Medical management of Cushing's disease – when and how?’ in this special issue.

**TABLE 3:** Summary of medical treatment used for the management of Cushing's syndrome in pregnancy

Drug	Benefits	Risks and limitations
Metyrapone^31^, ^35^, ^70^, ^74^, ^75^, ^76^, ^78^	Quick onset of action	Increased 11‐deoxycorticosterone increases risk of hypertension, pre‐eclampsia, oedema, hypokalaemia Not available universally Crosses placenta – may affect foetal adrenal steroidogenesis
Cabergoline^80^, ^82^, ^83^, ^84^	Evidence from pregnant women with prolactinoma to support its safety in pregnancy	Disrupts lactation Only four cases, two for which other treatment was used Limited efficacy when used outside of pregnancy
Ketoconazole^6^, ^70^, ^74^, ^79^, ^80^, ^81^	Efficacious outside of pregnancy	Teratogenic in animal studies (not seen in humans) Potential for foetal feminisation in males in first trimester Risk of severe liver injury
Cyproheptadine^87^, ^88^, ^89^	Use not recommended	Low efficacy Hyperphagia, weight gain, somnolence reported No longer recognised as a treatment for Cushing's syndrome
Mitotane^85^, ^86^	Use not recommended	Teratogenic
Aminoglutethimide^6^	Use not recommended	Foetal masculinisation

#### 7.3.1 Metyrapone

Outside of pregnancy, metyrapone, an 11‐ß hydroxylase inhibitor, has been shown to control cortisol in 76% of patients with CS^91^ and 75% of patients with CD.^92^, ^93^ Even with the application of strict definition criteria (mean serum cortisol level between 150–300 nmol L^−1^ assessed serially on a cortisol day curve), over 50% of patients with CS achieve target cortisol levels with metyrapone monotherapy.^94^

The evidence for use during pregnancy is limited to a small number of case reports,^31^, ^35^, ^70^, ^74^, ^75^, ^76^, ^77^, ^78^ but has been the medical treatment most commonly used in pregnancy.^30^ Of particular benefit, onset of action is rapid, with normalisation of urinary cortisol within the first few weeks.^92^ Given the elevated UFC values seen in healthy pregnancy, target UFC levels on metyrapone should sit around 1.5× the upper limit of normal.^64^ Treatment escape may be a limitation for some, though this usually occurs with increasing duration of treatment, and thus may not be of significance for those diagnosed later in pregnancy or if used as a bridging measure prior to surgery. Notable side effects include hypertension (secondary to 11‐deoxycorticosterone accumulation), hypokalaemia and oedema. Blood pressure should be monitored closely, particularly given the increased risk of pre‐eclampsia reported in active CS,^30^ in addition to close blood electrolyte monitoring. Access may be an issue for some countries.^95^ Metyrapone has been shown to cross the placenta in animal studies and, very recently, in one case report on a human subject.^75^ There is therefore the potential for foetal adrenal insufficiency. To date, no specific congenital malformations related to its use have been reported.

#### 7.3.2 Ketoconazole

Ketoconazole inhibits multiple enzymes involved in several stages of adrenal steroidogenesis.^96^ Outside of pregnancy, it has been shown to result in remission in 71% of patients with CS.^91^, ^95^

In animal studies, ketoconazole has been shown to be teratogenic and associated with an increased risk of abortion.^55^ Nevertheless, it has been used to good effect both in adrenal and pituitary Cushing's during pregnancy, without subsequent compromise to mother or foetus.^70^, ^74^, ^79^, ^80^, ^81^ If selected as a treatment option, it should be avoided during the first trimester given the risk of feminisation in males,^97^ although this was not seen in one case where a male infant was born following ketoconazole use for CD in the first trimester.^80^

#### 7.3.3 Cabergoline

There is a large body of evidence to support the safe use of dopamine agonists, with more than 6000 reports of bromocriptine and 1000 cases of cabergoline use during pregnancy for prolactinoma.^98^ The risk of adverse outcomes, including congenital malformation, is not greater compared to the general population.^3^ Safety outcomes have not been shown to differ between bromocriptine or cabergoline; thus, cabergoline is the dopamine agonist of choice given its superior efficacy and tolerability.^3^

Currently, there is insufficient evidence to recommend cabergoline use in CD during pregnancy. Given it acts directly on the pituitary, it is not suitable for patients with ACTH‐independent CS. Its efficacy may be limited, as remission of CD is reported in approximately 40% of patients treated outside of pregnancy.^99^ To date, there are just four case reports of cabergoline used during pregnancy for the management of CD, either used alone,^82^, ^83^, ^84^ or in combination with other medications.^80^ All cases were associated with good outcomes for both mother and child, although lactation was disrupted. Given that one patient had prior radiotherapy,^84^ and another had used ketoconazole concomitantly,^80^ the direct effects of cabergoline on disease activity are not clear.

#### 7.3.4 Other medical treatments

Cyproheptadine, a first‐generation antihistamine used historically in the management of CD, has been offered in a small number of cases during pregnancy.^87^, ^88^, ^89^ It is no longer a recognised treatment for CD as a result of a lack of efficacy^6^, ^100^ and is associated with a number of side effects, including hyperphagia, weight gain, and somnolence.^101^

Mifepristone and mitotane, comprising other potential medical treatments recognised in the management of CS, should not be considered in pregnancy given the risk of abortion and teratogenic potential.^6^

To the best of our knowledge, there are no reported cases of pariseotide or osilodrostat use for CS during pregnancy.

### 7.4 Adrenalectomy

Both unilateral and bilateral adrenalectomy have been performed during pregnancy, most with reasonably good outcomes.^9^, ^39^, ^78^, ^102^, ^103^, ^104^ Adrenal surgery is ideally recommended in the second trimester, but some advocate its use even in the third trimester.^105^, ^106^ Perinatal morbidity and mortality are improved, although premature delivery and intrauterine growth restriction (IUGR) do not appear to be influenced by unilateral adrenalectomy.^105^

Despite very limited evidence, some have recommended surgical ablation as the management of choice for ACC during pregnancy, regardless of gestational period.^107^ Unfortunately, both maternal and foetal outcomes for ACC are poor, and mitotane use for such cases is not recommended given its teratogenicity.^107^

Finally, pregnancy following bilateral adrenalectomy does not appear to promote tumour growth or accelerate Nelson's syndrome.^108^

## 8 MATERNAL OUTCOMES

As covered by Braunet al.^109^ in ‘Long term morbidity and mortality in patients with Cushing's syndrome’ in this special issue, active disease is associated with poorer outcomes. In pregnancy, adverse maternal outcomes are seen in > 50% of patients with active CD,^4^ highlighting the importance of pre‐conception counselling and of pregnancy avoidance in active disease. Two large reviews (including data on 263^5^ and 136^6^ pregnancies in women with CS) reported multiple associated complications; hypertension (40–68%), gestational diabetes (25–37%), pre‐eclampsia (14–27%), osteoporosis (5%), psychiatric disorders (4%), heart failure (3%), wound infections (2%), and maternal death (2%).^5^, ^6^, ^30^ The need for blood transfusion and assisted vaginal delivery are additional complications recently reported to be more likely in those with CS compared to those without.^110^ Maternal mortality in active disease is increased: Caimari et al.^5^ reported a maternal mortality ratio of 1257 per 100,000 population (6‐fold higher than that worldwide in 2013). Maternal outcomes in CS during pregnancy are shown in Tables 4 and 5.

**TABLE 4:** Risk of selected maternal complications associated with active Cushing's syndrome during pregnancy (data from systematic review performed by Caimari et al.^5^)

Maternal outcomes in Cushing's syndrome during pregnancy (active only)	
Maternal outcome variable	Percentage affected
Pre‐eclampsia	26.3%
Gestational hypertension	40.5%
Gestational diabetes mellitus	36.9%
Caesarean section delivery	51.7%

**TABLE 5:** Risk of selected maternal complications in patients with Cushing's disease during, or 1 year following pregnancy (as per systematic review by Sridharan et al.^4^)

Maternal outcomes in Cushing's disease during pregnancy (whole cohort^a^)	
Maternal outcome variable	Percentage affected
Pre‐eclampsia	21.2%
Gestational hypertension	19.1%
Gestational diabetes mellitus	21.2%
Caesarean section delivery	42.1%

^a^ Includes both treated and non‐treated patients with active disease.

When compared with those cured of CS by time of pregnancy, women with active disease are significantly more likely to develop gestational diabetes, gestational hypertension, and pre‐eclampsia, as well as to need a caesarean section.^5^ Those on treatment still face an increased risk of maternal morbidity. In the development of the 2021 European Society of Endocrinology clinical practice guidelines on the management of non‐functioning and functioning pituitary adenomas in pregnancy,^3^ a large proportion of mothers on medical treatment for CD had complications (60%; 95% confidence interval = 26%–88%), including gestational diabetes mellitus, hypothyroidism, pre‐eclampsia, and disrupted lactation. A systematic review by Sridharan et al.,^4^ which focussed on those with CD in pregnancy only, found no difference in the rate of maternal adverse outcomes when comparing those who received treatment (medical or surgical) with those who were conservatively managed, although the numbers were too small to allow statistical comparison for individual outcomes.^4^ Similarly, in a recent retrospective cohort study exploring the risk of maternal and foetal comorbidities in 60 patients (78 pregnancies) with CD diagnosed and managed in tertiary referral centres before or during pregnancy, the prevalence of some maternal complications, including hypertension and pre‐eclampsia, did not differ between eucortisolaemic and hypercortisolaemic patients. The small number of cases, differences in treatment period and subsequent advances in obstetric management, as well as the possibility of milder disease in those conservatively managed, may account for the lack of significant difference between those treated compared to those not treated.^4^, ^111^ Nevertheless, in the latter study,^111^ the prevalence of maternal complications in eucortisolaemic patients was comparable to maternal risk in the background population. This is in concordance with conclusions from other studies in which those who have been cured of CS tend to normalise their risks of maternal complications.^3^, ^5^, ^111^

## 9 FOETAL OUTCOMES

The risk of foetal adverse events is increased irrespective of whether a mother with CS in pregnancy is treated medically, surgically or not at all.^3^ Such complications include foetal death, pre‐term delivery, IUGR, foetal and respiratory distress, low birth weight, and foetal hypoadrenalism.^4^, ^5^, ^6^, ^7^ Foetal loss has been reported in 12.5%, 20.8%, and 30.6% of cases, in those who were managed surgically, medically or untreated, respectively.^3^, ^7^ Despite these alarming statistics, the risk of foetal death in pregnant patients with CS in remission is comparable to that of the healthy population, and almost 3‐fold less likely, compared to those with active CS.^5^ Pregnancy‐induced hypercortisolism, treatment during pregnancy, and earlier treatment era have been identified as risk factors associated with foetal loss in those with active CS.^5^

Beyond foetal death, those with active CS have a higher risk of perinatal death, foetal distress, premature birth (delivery prior to 37 weeks), low birth weight and respiratory distress compared to those in remission.^5^ However, in contrast to foetal loss, no significant difference in risk of prematurity,^5^, ^6^, ^105^, ^111^ IUGR^6^ or low birth weight,^5^ has been demonstrated when comparing those treated medically or surgically with those conservatively managed. Indeed, over half of infants (58%; 95% confidence interval = 28%–85%) of mothers with CD treated with medical therapy experienced adverse events including neonatal death, low birth weight, premature delivery, small for gestational age or a need for intensive care according to the European Society for Endocrinology clinical practice guidelines.^3^ However, such statistics arise from low quality evidence, from just 10 mothers and 12 infants (two pairs of twins).^3^ Comparisons between treated and non‐treated groups are limited by differences in baseline characteristics (such as severity of hypercortisolaemia or number of complications) which, in turn, may have influenced intervention type and confound the outcomes. Of greater reassurance, the majority of foetal outcomes in those with disease in remission appear comparable to those recorded in the healthy population^5^ and, to date, no specific congenital malformation as a result of CS has been identified.^3^

## 10 POST‐PARTUM

### 10.1 Reassessment of HPA axis

Reassessment of the HPA axis is recommended at 2–3 months post‐delivery.^3^ In those with untreated CD, pituitary MRI should be performed 3–6 months after birth.^55^

### 10.2 Hypoadrenalism following treatment for CS

Although the management of hypoadrenalism in pregnancy is beyond the scope of this review, many patients treated for CS will develop secondary hypoadrenalism, either transiently or permanently, and may go on to have subsequent pregnancies. Similar to all with adrenal insufficiency, such patients should be advised to increase their glucocorticoid replacement dose by 20%–40% from week 22 onwards, aiming to mimic the rise in free cortisol during this period.^3^ For all with hypoadrenalism, i.v. stress doses of hydrocortisone during the second stage of labour or before the onset of caesarean delivery are necessary, and double dosing of usual physiological doses is usually warranted for 2–3 days following delivery.^112^ Education on sick day rules is essential, particularly given that commonly encountered manifestations (such as hyperemesis) may simply be attributed to pregnancy, and lead to adverse sequelae if not appropriately managed.

### 10.3 Preconception counselling

Although relevant to all women of reproductive age with CS, contraception should be started soon in the post‐partum period and active disease has to be addressed prior to any consideration of further pregnancies. Women with active disease should be advised to avoid pregnancy, explaining the potential challenges and risks in the event of becoming pregnant.^3^ Given the increased risk of thrombosis in those with CS, oestrogen‐progestin oral contraceptives should be avoided, and non‐hormonal contraception be advised.^55^, ^73^

## 11 CONCLUSIONS

**FIGURE 3:**
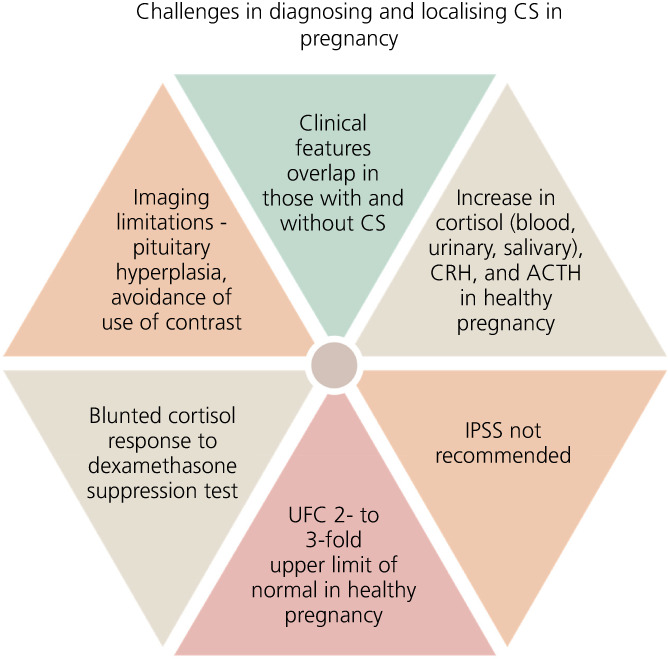
Schematic highlighting the challenges associated with a diagnosis of Cushing's syndrome (CS) in pregnancy. Abbreviations: ACTH, adrenocorticotrophic hormone; CRH, corticotrophin‐releasing hormone; IPSS, inferior petrosal sinus sampling; UFC, urinary free cortisol

CS in pregnancy is extremely rare and challenging to diagnose (Figure 3). In the presence of many overlapping clinical and biochemical features encountered in both healthy pregnancy and in those with CS, a high index of suspicion is necessary.

For diagnosis, measurements of UFC and LNSC are the most helpful tools. For the differential diagnosis, ACTH above, or in the higher end of the reference range, points towards ACTH‐dependent CS, whereas values below these may also be seen in adrenal CS. Imaging (pituitary or adrenal MRI without contrast) will guide further steps.

Maternal and foetal complications are broad, and those with active disease carry the greatest risk. Management should be individualised, conducted by a multidisciplinary team in a tertiary centre with expertise in high‐risk pregnancies. With the caveat of limited evidence, surgical management (TSS or adrenalectomy dependant on aetiology) should be considered for those with moderate to severe hypercortisolaemia, ideally in the second trimester of pregnancy. For those outside this gestational window, or in those with milder disease, medical treatment needs to be considered. In selected cases of mild disease where complications are controllable by supportive medications, close monitoring alone may be sufficient. Regardless of treatment approach, low molecular weight heparin should be considered for all, given the significantly elevated risk of thrombosis.

Following delivery, the HPA axis can be reassessed at 2–3 months. Pre‐conception counselling has to be given to all women with CS who are of child‐bearing age, and contraception methods (without associated thrombotic risk) should be strongly recommended in those with active CS.

This article is part of an update series on the diagnosis and treatment of Cushing's syndrome.^90^, ^109^, ^113^, ^114^, ^115^, ^116^, ^117^, ^118^, ^119^, ^120^, ^121^, ^122^, ^123^, ^124^, ^125^, ^126^, ^127^

## CONFLICT OF INTEREST

The authors declare that they have no conflicts of interest.

## AUTHOR CONTRIBUTIONS

**Ross Hamblin:** Writing – original draft. **Amy Coulden:** Writing – original draft. **Athanasios Fountas:** Writing – review and editing. **Niki Karavitaki:** Supervision; writing – review and editing.

### PEER REVIEW

The peer review history for this article is available at https://publons.com/publon/10.1111/jne.13118.

## Data Availability

Data sharing is not applicable to this review because no new data were created or analyzed.
